# Pronounced capping effect of olaminosomes as nanostructured platforms in ocular candidiasis management

**DOI:** 10.1080/10717544.2022.2120926

**Published:** 2022-09-07

**Authors:** Sadek Ahmed, Maha M. Amin, Sarah Mohamed El-Korany, Sinar Sayed

**Affiliations:** a Department of Pharmaceutics and Industrial Pharmacy, Faculty of Pharmacy, Cairo University, Cairo, Egypt; b Department of Microbiology and Immunology, Faculty of Pharmacy, Cairo University, Cairo, Egypt

**Keywords:** Fenticonazole nitrate, olaminosomes, corneal uptake, Minimal Inhibitory Concentration, *ex vivo* corneal penetration, corneal tolerance

## Abstract

The aim of this study was to formulate and boost ocular targeting of Fenticonazole Nitrate (FTN)-loaded olaminosomes in order to improve drug corneal permeation and candidiasis treatment. Olaminosomes were formulated by ethanol injection technique applying a central composite design. The independent variables were: span 80 amount (mg) (A), oleylamine concentration (mg%) (B) and oleic acid: drug ratio (C). The dependent responses were: percent entrapment efficiency (EE %), particle size (PS), poly-dispersity index (PDI), zeta potential (ZP) and *in vitro* drug release after 10 hours (Q10h). Numerical optimization by Design-Expert® software was adopted to select the optimum formula. This formula was chosen based on highest EE %, ZP (as absolute value) and Q10h and lowest PS and PDI. The optimum formula was subjected to further *in vitro* characterization via Differential scanning calorimetry, Transmission electron microscopy, Fourier transform infrared spectroscopy, pH determination, effect of storage, influence of terminal sterilization, detection of Minimal Inhibitory Concentration and *ex vivo* corneal penetration analysis. Safety and antifungal activity of the optimum formula were tested through various *in vivo* studies like ocular irritancy, corneal tolerance, corneal uptake and susceptibility test. The optimum formula with the maximum desirability value (0.972) revealed EE% (84.24%), PS (117.55 nm), ZP (−74.85 mV) and Q10h (91.26%) respectively. The optimum formula demonstrated ocular tolerance with enhanced corneal penetration behavior (428.66 µg/cm^2^) and boosted antifungal activity (56.13%) compared to FTN suspension (174.66 µg/cm^2^ and 30.83%). The previous results ensured the ability of olaminosomes to enhance the corneal penetration and antifungal efficacy of Fenticonazole Nitrate.

## 1. Introduction

Eye is a very delicate organ with a complicated structure composed of anterior and posterior segments. Generally, quality of life is considerably affected by visual impairment caused from several diseases such as cataract, glaucoma, age-related macular degeneration, diabetic retinopathy, retinoblastoma and ocular candidiasis (Krishnaswami et al., 2018). Fungal ocular diseases are among microbial infections that might be considered as chief causes of blindness worldwide, principally in developing countries. *Candida albicans* causes candidiasis that represents the most dangerous fungal infection which influences the public health. Moreover, candidiasis could be acute or chronic and superficial or deep (Mohsen, 2022).

As a general rule, the overuse of conventional antimicrobial medications results in development of microbial resistance, therapy loss of their effectiveness (Naz et al., 2018). Furthermore, some antimicrobial medications suffer from certain side effects such as their toxicity or irritation that necessitate development of novel delivery systems in order to fabricate safe and cost-effective treatment. Although different nanosystems are used in ocular delivery of antimicrobial medications, the main challenge is to formulate a dosage form achieving the required shape and size (Ali et al., 2020). Consequently, fabrication, *in vitro* characterization, *ex vivo* and *in vivo* assessments are the key steps to develop innovative generation of ocular antimicrobial medications (Sondi & Salopek-Sondi, 2004). The major drawbacks of various nanosystems are aggregation due to instability, toxicity, size and shape imperfections, therapy surface modification was employed. Many researches encourage the positive consequences of surface modification on enhancing the safety and efficacy of developed nanosystems.

Capping agents are of chief significance as stabilizers that prevent the over-growth and agglomeration of the developed nanoparticles. Capping agents have particular structural properties that stabilize the interface of nanoparticles, change their biological behaviors and alter their environmental perception. Steric hindrance of capping agents resulted from the developed covalent bonds between the chains of capping agents and the interface of the nanoparticles. Generally, reducing the size into nanoscale will increase the percentage of atoms at the surface, therapy capping effects enhances (Javed et al., 2020).

Recently capping agents are used in frequent to control the growth, prevent aggregation and maintain the physicochemical characteristics in a defined manner (Niu & Li, 2014). Capping agents are amphoteric molecules that are composed of hydrophilic head and lipophilic tail, hence they can boost the compatibility and functionality with alternative phase. There are many capping agents that are used in pharmaceutical industry such as surfactants, small ligands, polymers, dendrimers, cyclodextrins, and polysaccharides. These capping agents succeed to provoke delicate modifications in nanoparticles causing remarkable therapeutic effects (Radini et al., 2018). Therefore, the selection of appropriate capping agent is fundamental in stabilizing the developed nanosystem and regulating their uptake into living cells and the environment. Capping agent alter the surface chemistry and size distribution of the formulated nanoparticles (Javed et al., 2020). In this study, oleylamine was selected as capping agent as it has the capability to form a carboxylate derivatives with the carboxylic group of oleic acid (Safo et al., 2019). Oleylamine is a long-chain primary alkyl amine that utilizes its amine group (NH_2_-) for interaction. As, amine group has greater affinity for protons resulted from the lone pair of electrons it owns which it simply donates to H. Oleylamine has a high proton that allows it to interact easily (Mbewana-Ntshanka et al., 2020).

Azoles represents a major class of antifungal drugs that act as fungistatic at low concentration by inhibiting ergosterol synthesis, however at high concentration they have a fungicidal activity by destroying the fungal cell wall (Chaudhari et al., 2022). Fenticonazole nitrate (FTN) belongs to the antifungal imidazole family with poor water solubility less than 0.10 mg/mL. Previous nanoparticles of FTN were formulated including terpesomes (Albash et al., 2021) and cerosomes (Albash et al., 2021). Albash et al., developed ocular FTN-loaded terpesomes with enhanced residence time and drug permeation (Albash et al., 2021). Topical cerosomal systems of FTN were also formulated with enhanced localization, retention tendency and antifungal efficacy (Albash et al., 2021).

Precisely, the aim of our investigation is to formulate and develop ocular oleylamine capped FTN-loaded olaminosomes adopting ethanol injection method. Analysis of the independent variables was carried out via central composite design (CCD), by examining the effect of span 80 amount (mg), oleylamine concentration (mg%) and oleic acid: drug ratio on percent entrapment efficiency (EE %), particle size (PS), poly-dispersity index (PDI), zeta potential (ZP), and *in vitro* drug release after 10 hours (Q10h). The FTN-loaded optimum formula was subjected to further *in vitro* characterization concerning (DSC, FTIR, TEM), pH determination, effect of storage, influence of terminal sterilization, detection of Minimal Inhibitory Concentration (MIC). Penetration parameters were investigated via *ex vivo* corneal penetration study. Safety of the FTN-loaded optimum formula was verified though *ex vivo* corneal hydration level, *in vivo* ocular irritancy test and *in vivo* corneal tolerance test. Finally, efficacy of the FTN-loaded optimum formula was investigated through determination of *in vivo* corneal uptake and susceptibility test.

## 2. Materials and methods

### 2.1. Materials

FTN was supplied as a gift from Andalous Pharmaceutical Co. (Cairo, Egypt). Oleylamine, span 80, methanol (HPLC grade), dialysis membrane (typical molecular weight cutoff 14,000 Da) and Rhodamine B were purchased from Sigma Chemical Company. Ethanol (95%), isopropyl alcohol and formaldehyde were supplied from El-Nasr pharmaceutical chemicals Co. (Cairo, Egypt). Other chemicals and solvents were of analytical grade and were utilized without any further purification.

### 2.2. Animals

Adult male albino rabbits, having an average body weight of 2 ± 0.5 kg, were housed individually (one per cage) at 25 ± 2 °C, with 12 hours cycle alternating of light and dark. Animals were supplied with the standard commercial food and tap water. Initial examination of all rabbits’ eyes was carried out. Only the rabbits with no signs of ocular inflammation were included in the study. Approval of animal procedures was obtained from the Research Ethics Committee for experimental and clinical studies of Faculty of Pharmacy, Cairo University, Egypt (Approval no. PI 3132) and was performed conforming the Guide for Care and Use of Laboratory Animals announced by the US National Institute of Health (NIH Publication No. 85–23, revised 2011).

### 2.3. Methods

#### 2.3.1. Statistical design for FTN-loaded olaminosomes

Central composite design was used to formulate FTN-loaded olaminosomes. This design includes twenty trails which are: 8 factorial points, 6 axial points and 6 replicated center point. Alpha was placed at 1.68179. The studied experimental factors were: span 80 amount (mg) (A), oleylamine concentration (mg%) (B) and oleic acid: drug ratio (C) all at three levels. Election of these levels was based on preliminary findings and they were defined as (−1, 0, +1). Their resembling values are shown in Table 1. The researched responses were: percent entrapment efficiency (EE %) (Y1), particle size (PS) (Y2), poly-dispersity index (PDI) (Y3), zeta potential (ZP) (Y4), and *in-vitro* release after 10 hours (Q10h) (Y5). Significance of each factor was studied using Design-Expert^®^ software version 7 (Stat-Ease, Inc., Minneapolis, Minnesota, USA) (Ahmed et al., 2020).

**Table 1.** Factorial levels of investigated independent variables in the central composite design together with measured responses and their desirability constraints.

	Level		
Factors (independent variables)	−1	0	+1
A: Span 80 amount (mg)	7.5	15	22.5
B: Oleylamine concentration (mg%)	25	50	75
C: Oleic acid: drug ratio	5:1	10:1	15:1
Responses (dependent variables)	Desirability constraints		
Y1: EE %	Maximize		
Y2: PS (nm)	Minimize		
Y3: PDI	Minimize		
Y4: ZP (absolute value) (mV)	Maximize		
Y5: Q10h (%)	Maximize		

Abbreviations: EE %, percent entrapment efficiency; PDI, poly-dispersity index; PS, particle size; Q10h, percent drug released after 10 hours; ZP, zeta potential.

#### 2.3.2. Preparation of FTN-loaded olaminosomes

Olaminosomes of FTN were constructed by ethanol injection technique (Gouda et al., 2021) with little alteration. Concisely, FTN (10 mg), Specified amount of oleic acid, span 80 and oleylamine were exactly weighted and dissolved with (10 mL) ethyl alcohol in a water bath at 60 °C. Then, transferred slowly into a four-time larger volume of phosphate-buffered saline (PBS, pH 7.4) that was maintained and stirred at the same temperature till the complete disappearance of ethyl alcohol. Olaminosomes formation was noticed by the appearance of abrupt turbidity. Afterwards, the resultant dispersion was sonicated at 25 ± 2 °C to minimize the PS of the prepared olaminosomes and the resulted formulae were preserved at 4 °C till further assements (Al-Mahallawi et al., 2014). The trials (T1-T20) are prepared and characterized according to the random order of Table 2.

**Table 2.** Composition of the various formulated FTN-loaded olaminosomes with their measured responses of central composite design (*n* = 3 ± SD).

	Factors			Responses				
Trial	A: Span 80 amount (mg)	B: Oleylamine concentration (mg%)	C: Oleic acid: drug ratio	Y1: EE % (Mean +SD)	Y2: PS (nm)(Mean +SD)	Y3: PDI(Mean +SD)	Y4: ZP (mV) (Mean +SD)	Y5: Q10h (%)(Mean +SD)
T1	2.39	50	10:1	53.33 ± 1.96	348.85 ± 0.64	0.08 ± 0.10	−54.40 ± 3.11	84.08 ± 3.17
T2	7.5	25	5:1	55.56 ± 3.34	356.60 ± 8.49	0.29 ± 0.18	−30.20 ± 1.84	82.24 ± 1.96
T3	7.5	25	15:1	58.47 ± 2.55	160.70 ± 4.67	0.20 ± 0.01	−70.90 ± 1.27	82.88 ± 2.27
T4	7.5	75	15:1	63.47 ± 1.18	159.55 ± 2.33	0.12 ± 0.06	−72.20 ± 0.14	92.99 ± 2.94
T5	7.5	75	5:1	63.89 ± 4.71	197.45 ± 1.20	0.22 ± 0.01	−36.65 ± 0.78	92.09 ± 2.24
T6	15	50	10:1	66.11 ± 0.98	185.85 ± 1.91	0.23 ± 0.03	−57.95 ± 0.21	86.29 ± 2.47
T7	15	50	10:1	66.25 ± 1.57	174.55 ± 1.34	0.21 ± 0.01	−59.55 ± 1.06	87.06 ± 2.52
T8	15	50	10:1	67.08 ± 1.57	173.20 ± 0.42	0.22 ± 0.01	−61.55 ± 1.77	87.45 ± 2.28
T9	15	50	10:1	68.06 ± 1.77	171.45 ± 1.34	0.40 ± 0.01	−61.55 ± 1.77	88.37 ± 2.24
T10	15	7.96	10:1	68.89 ± 2.36	171.20 ± 7.78	0.30 ± 0.01	−46.40 ± 3.96	80.37 ± 2.49
T11	15	50	10:1	70.00 ± 0.79	170.40 ± 0.71	0.21 ± 0.00	−61.85 ± 2.19	88.75 ± 2.35
T12	15	50	10:1	73.19 ± 0.98	167.70 ± 5.52	0.38 ± 0.06	−64.40 ± 0.14	90.70 ± 3.31
T13	15	50	1.59:1	73.89 ± 2.16	427.35 ± 78.14	0.41 ± 0.03	−27.10 ± 0.85	85.65 ± 2.19
T14	15	92.04	10:1	80.14 ± 2.16	156.85 ± 1.06	0.14 ± 0.02	−68.00 ± 0.57	94.44 ± 2.61
T15	15	50	18.41:1	80.42 ± 1.37	159.20 ± 5.09	0.19 ± 0.03	−73.80 ± 4.38	90.83 ± 2.04
T16	22.5	25	5:1	81.46 ± 3.44	192.80 ± 0.28	0.24 ± 0.03	−31.25 ± 2.47	83.53 ± 1.89
T17	22.5	75	15:1	82.50 ± 1.57	152.20 ± 2.26	0.30 ± 0.03	−73.60 ± 2.40	93.57 ± 2.82
T18	22.5	25	15:1	83.47 ± 0.98	149.00 ± 0.42	0.14 ± 0.01	−70.90 ± 1.27	84.40 ± 2.38
T19	22.5	75	5:1	87.99 ± 2.26	188.50 ± 1.84	0.25 ± 0.00	−43.85 ± 0.35	93.34 ± 2.73
T20	27.61	50	10:1	88.26 ± 0.29	143.00 ± 2.26	0.16 ± 0.00	−66.05 ± 2.19	90.66 ± 2.41

Abbreviations: EE %, percent entrapment efficiency; PDI, poly-dispersity index; PS, particle size; Q10h, percent drug released after 10 hours; ZP, zeta potential.

#### 2.3.3. *In vitro* characterization of the prepared FTN-loaded olaminosomes

##### 2.3.3.1. Percent entrapment efficiency (EE %)

FTN-loaded olaminosomes were separated from the free FTN (unentrapped FTN) by centrifugation (3K30, Sigma, Germany) at 21,000 rpm for 1 hour at 4 °C. Spectrophotometer (Shimadzu, model UV-1601 PC, Kyoto, Japan) was utilized to detect the concentrations of FTN in the supernatant at the predetermined λmax 252 nm employing the calibration curve (*n* = 3, R^2^= 0.9998). The EE % was estimated using the subsequent formula (Elsayed & Sayed, 2017; Sayed et al., 2020):

(1) $$EE\;\%\;=\frac{\left(\text{total amount of FTN}-\text{ total amount of free FTN}\right)}{\text{total amount of FTN}}\;\text{X }100$$

Total amount of FTN is the actual amount used, total amount of free FTN (quantity of FTN in supernatant)

##### 2.3.3.2. Particle size (PS), poly-dispersity index (PDI) and Zeta-potential (ZP)

Photon correlation spectroscopy was used to assess PS, PDI and ZP of the formed olaminosomes. Concisely, 1 mL of each formula was diluted 10 times with distilled water till being translucent. Then, Malvern Zetasizer (Model ZEN3600, Malvern Instruments Ltd. Worcestershire, UK) was used to perform the measurements at 25 °C (Ahmed et al., 2021). Mean of three repetitive results were taken as average ± SD.

##### 2.3.3.3. *In vitro* release studies

Bag dialysis method (typical molecular weight cutoff 14,000 Da; Sigma-Aldrich Co.) was used to detect the *in vitro* release profiles of FTN from the formulated olaminosomal dispersions. Succinctly, dialysis membrane was dipped overnight in the release medium (ethanoic phosphate buffer saline solution (25%v/v, pH 7.4 to maintain the sink condition) (Elsayed & Sayed, 2017). Then, a dialysis bag containing 2 mL (equivalent to 0.5 mg of FTN) of either the prepared formula or FTN suspension was soaked in amber glass bottles containing 25 mL of the release medium (Albash et al., 2021). Glass bottles were positioned in a thermostatically controlled shaker working at 37 ± 0.5 °C and at 100 rpm. At predetermined intervals of (0.5, 1, 2, 4, 6, 8, 10 h), samples of 3 mL were removed and substituted rapidly with equal volume of fresh medium in to continue sink condition. The percent released was estimated by spectrophotometric measurement at λmax 252 nm using the predetermined calibration curve (*n* = 3, R^2^= 0.9992) and plotted against time. All release profiles were tailored to zero, first, and Higuchi diffusion models. The best model is the one with largest coefficient (R^2^) (Ahmed et al., 2020).

#### 2.3.4. Optimization and validation

All the resulted responses were evaluated via analysis of variance (ANOVA) using Design-Expert^®^ software version 7 (Stat-Ease, Inc., Minneapolis, Minnesota, USA). The FTN-loaded optimum formula was elected in terms of desirability to instantaneously ensure the possible highest EE %, ZP (as absolute value), Q10h and the lowest PS and PDI. To ensure the validity of statistical models, the FTN-loaded optimum formula was formulated, exposed to the formerly stated characterizations, and the outcomes were matched to predicted values with estimating the deviation percentages (Ahmed et al., 2021; Said et al., 2021).

#### 2.3.5. *In vitro* evaluation of the FTN-loaded optimum formula

##### 2.3.5.1. Differential Scanning Calorimetry (DSC)

Firstly, the optimum formula was freezed at (−20 °C) then dried at (−45 °C) under lowered pressure for (24 h) (Novalyphe-NL 500 freeze-dryer, Savant Instruments, NY, USA) (Sayed et al., 2018). The thermal properties of pure FTN, oleylamine, lyophilized FTN-loaded and lyophilized FTN-free optimum formula were detected via differential scanning calorimetry DSC7 (Perkin-Elmer, Waltham, MA) standardized with indium. Around 2 mg from each formula was positioned in aluminum pan and heated to 300 °C at a rate of 5 °C/min under nitrogen stream (25 mL/min) (Albash et al., 2020).

##### 2.3.5.2. Fourier transform infrared spectroscopy (FTIR)

FTIR spectra of pure FTN, oleylamine, lyophilized FTN-loaded and lyophilized FTN-free optimum formula were determined by FTIR spectrophotometer (model 22, Bruker, Coventry, UK). The samples were formulated in potassium bromide (KBr) pellets and their spectrum was gained in the range between 4000 and 500 cm^−1^ at 25 ± 2 °C (Younes et al., 2018a).

##### 2.3.5.3. Transmission electron microscopy (TEM)

Morphology of the FTN-loaded optimum formula was evaluated by TEM (JEOL, Tokyo, Japan). Briefly, one drop from the FTN-loaded optimum formula dispersion was placed as a thin film on a carbon laminated copper grid, left to dry then stained using phosphotungstic acid 2% w/v (Younes et al., 2018b).

##### 2.3.5.4. pH measurement

The pH of the FTN-loaded optimum formula was determined by a pH meter (model-3505, Jenway, Staffordshire, UK). pH determination is important to verify the suitability and efficacy of the FTN-loaded optimum formula (Fahmy et al., 2021; Sayed et al., 2021). As a General rule, alkaline(pH > 10) or acidic (pH < 4) solutions are harmful to the eye (Said et al., 2021). In addition, enhanced ocular penetration was noticed when pH varies from 4 to 8 (Mohanty et al., 2013). The pH of ophthalmic product is usually varied from 3.50 to 8.50 (Said et al., 2021).

##### 2.3.5.5. Influence of short-term storage

Generally, Short-term storage study was performed to confirm the ability of ocular products to preserve their properties and efficacy after storage under certain conditions (Albash et al., 2021). The FTN-loaded optimum formula was stored at (4–8 °C) for three months. Then, it was re-assessed at the end of storage period concerning its physical appearance, EE %, PS, ZP and Q10h compared to the freshly formulated formula (Al-Mahallawi et al., 2017). EE %, PS, ZP were matched using one-way ANOVA test. However, similarity factor ‘ƒ_2_’ was used to compare the release profiles of the fresh and stored FTN-loaded optimum formula. Similarity factor ‘ƒ_2_’ was determined by applying the following equation (Abd-Elbary et al., 2008):

(2) $$f_{2}=50.\log\{[1+(\frac{1}{n})\sum_{t=1}^{n}{\left(R_{t}-T_{t}\right)}^{2}{]}^{-0.5}.100$$

R_t_ and T_t_ are the % FTN released from the fresh and stored FTN-loaded optimum formula respectively at time t. Similarity factor ‘ƒ2’ value between 50 and 100 ensures the resemblance (Sayed et al., 2020; 2021).

##### 2.3.5.6. Influence of terminal sterilization

The FTN-loaded optimum formula was subjected to terminal sterilization using Cobalt-60 irradiator at rate of 1.774 kGy/h and radiation dose of 25 kGy in an Indian Gamma cell (Sayed et al., 2020). Reevaluation in terms of physical appearance, EE %, PS, ZP and Q10h was done to ensure the stability of the optimum dispersion. EE %, PS, ZP were matched using one-way ANOVA test. But, similarity factor ‘ƒ_2_’ was used to compare the release profiles before and after sterilization. R_t_ and T_t_ are the % FTN released from the FTN-loaded optimum formula before and after sterilization respectively at time t. Similarity factor ‘ƒ2’ value between 50 and 100 ensures the resemblance (Sayed et al., 2020; 2021).

##### 2.3.5.7. Minimum Inhibitory Concentration (MIC) determination

MIC was calculated using broth microdilution method following Clinical and Laboratory Standards Institute guidelines (Humphries et al., 2018). A volume of 150 μL of twofold strength Sabouraud dextrose broth (SDB) was added to the wells of a sterile U-shaped bottom 96-well plate. Another 150 μL of each of the tested formulae (FTN suspension and the FTN-loaded optimum formula) was added to the first well of each row. Then two-folds serial dilutions of each of the tested formulae were done from one row to the next one till reaching the tenth row (250–0.49 μg/mL). The wells were inoculated with 10 μL of *Candida albicans* ATCC 60193 suspension (10^7^ CFU/mL). Each row included one well as a negative control for sterility (neither yeast suspension nor tested formula was added) and another well as a positive control for growth (inoculated with yeast suspension only). Plates were incubated at 25 ± 2 °C for 24 hours in aerobic condition. MIC was the lowest concentration showing no observable microbial growth. The experiment was repeated at three independent times (Albash et al., 2021; Fahmy et al., 2021).

#### 2.3.6. *Ex vivo* characterization of the FTN-loaded optimum formula

##### 2.3.6.1. *Ex vivo* corneal penetration

The study was reviewed and accepted by Ethics Committee, Faculty of Pharmacy, Cairo University (PI 3132) and fulfilled with the Guide for Care and Use of Laboratory Animals published by the US National Institute of Health (NIH Publication No 85-23, revised 2011). Male albino rabbits (weight 2 ± 0.5 kg) were firstly anesthetized by intramuscular injection of 35 mg/kg ketamine and 5 mg/kg xylazine (Elsayed & Sayed, 2017; Emad Eldeeb et al., 2019). Later on, decapitation was done to separate the cornea and sclera that were cleaned by PBS (pH 7.4) and immediately connected to one end of the open ended tube containing 15 mL of the receptor medium (ethanoic phosphate buffer saline solution (25%v/v, pH 7.4 to maintain the sink condition). Donor medium (FTN-loaded optimum formula or FTN suspension) enclosed 0.5 mg of FTN.

Samples of 0.5 mL were removed at predetermined intervals of (1, 2, 4, 6, 8, 10 h) and substituted quickly with new receptor medium to maintain sink condition (Sayed et al., 2020). The collected samples were purified using 0.45 μm membrane filter and the penetrated drug was determined at each interval by HPLC (Shimadzu, Tokyo, Japan) functioned using RP-C18 column (250 × 4.6 mm, 5 μm) and UV detector at λmax (252 nm). Mobile phase of methanol-water (85:15, v/v run at consisted of) was filtered, degassed. The flow rate was 1.2 mL/min with an injection volume of 20 μL (Ahmed et al., 2022). The quantity of FTN penetrated per unit area (µg/cm^2^) was graphed against time (h). Cumulative amount of FTN penetrated through the corneal membrane per unit area after 10 hours (Q_10h-permeation_), flux after 10 hours (J_max_) and the enhancement ratio (ER) were estimated for the studied preparations. The flux (J_max_) and the enhancement ratio (ER) were assessed from the following equations (Sayed et al., 2018):

(3) $$J_{\max}\;=\frac{\mathrm{Amount}\;of\;\mathrm{drug}\;\mathrm{permeated}}{\mathrm{Time}\;X\;\mathrm{Area}}$$

(4) $$\mathrm{ER}\;=\;\frac{\mathrm{Jmax}\;of\;\mathrm{formulation}}{\mathrm{Jmax}\;of\;\mathrm{drug}\;\mathrm{suspension}}$$

##### 2.3.6.2. *Ex vivo* corneal hydration level

Following the ex vivo test, each cornea was removed, cleaned and delicately dried with a filter paper to get rid of the additional water and then weighed rapidly to detect wet corneal weight (W_w_). Later, it was kept at 50 °C for 24 h and reweighed to detect dry corneal weight (W_d_). One-way ANOVA was used to compare the corneal hydration levels (HL%) of the FTN-loaded optimum formula and FTN suspension. The corneal hydration level (HL %) was calculated using the next equation (Moustafa et al., 2017):

(5) $$HL\;\%=[1-(\frac{W_{d}}{W_{w}})].100$$

#### 2.3.7. *In vivo* characterization of the FTN-loaded optimum formula

##### 2.3.7.1. Ocular irritancy test

In order to ensure the safety of the FTN-loaded optimum formula, ocular irritancy test was conducted. Any possible harming outcomes from the FTN-loaded optimum formula was assessed by noticing any inflammation, sensitivity or increased tear production after administration of the FTN-loaded optimum formula. The test was achieved using three albino rabbits. The tested formula was administrated into one eye, while the other eye served as a control. Both eyes were tested for any mark of inflammation, for example conjunctival corneal edema and/or hyperhemia upon direct visual examination utilizing a slit lamp, afore treatment and 1, 8 and 24 h subsequent administration (Abdelbary et al., 2017).

##### 2.3.7.2. *In vivo* corneal tolerance

The biocompatibility of the FTN-loaded optimum formula was tested via histopathological study. The FTN-loaded optimum formula was tested and related to sterile normal saline (negative control) and isopropyl alcohol 95% (positive control). Three albino rabbits (weight 2 ± 0.5 kg) were used to perform the experiment. Concisely, one drop from of normal saline or isopropyl alcohol 95% was administrated into one eye of a male albino rabbit. While the FTN-loaded optimum formula was administrated into the second eye. Each liquid was administrated twice daily for one week (Abdelbary et al., 2016; Elsayed & Sayed, 2017). Rabbits were anesthetized as mentioned in the previous *ex vivo* study and corneas were removed from the decapitated animals and stored in 10% v/v formalin saline solution until monitored. Washing was done in tap water then serial dilutions of alcohol (methyl, ethyl and absolute ethyl) were used for dehydration. Specimens were cleared in xylene and embedded in paraffin at 56 °C for 24 h in hot air oven. Paraffin bees wax tissue blocks were prepared for sectioning at 4 microns thickness by Leitz rotary microtome. The obtained tissue sections were collected on glass slides, deparaffinized, stained by hematoxylin & eosin stain for examination through the light electric microscope (Younes et al., 2018a).

##### 2.3.7.3. *In vivo* corneal uptake

To carry out this test, FTN in the FTN-loaded optimum formula was replaced by 0.1% w/w Rhodamine B (RhB) to be visualized under Confocal laser scanning microscopy (CLSM) (LSM 710; Carl Zeiss, Jena, Germany). Briefly, the right eye of male albino rabbit (weight 2 ± 0.5 kg) received one drop (100 µL) of RhB-loaded formula, however the left RhB-loaded aqueous solution (negative control) was administrated into the left eye to serve as a control. After 6 h, the animals were sacrificed by decapitation as mentioned before and corneas were delicately separated, cleaned and preserved in artificial tears until imaging. RhB was fluorescently detected by excitation at 485 and 595 nm utilizing argon and helium–neon lasers, respectively. Confocal images were processed and rectified using LSM software version 4.2 (Carl Zeiss Microimaging, Jena, Germany) (Elsayed & Sayed, 2017).

##### 2.3.7.4. Susceptibility test

Six rabbits were divided randomly into 2 groups (3 rabbits in each group, *n* = 3) where group I received the FTN-loaded optimum formula and group II received FTN suspension. *Candida albicans* ATCC 60193 was used as the test organism. The experiment was performed as described by Basha et al., but with slight modifications (Basha et al., 2013). Briefly, fifty microliters of each of the tested formulae (FTN suspension and the FTN-loaded optimum formula) were administered within the lower conjunctival sac of the right eye of each rabbit using a micropipette. No drug was inserted in the left eye of each rabbit to serve as a control. At specific time intervals (1–10 hours), four sterile filter paper disks (Whatman no. 5, 6 mm in diameter) were moistened by placing the disks under the eyelid of each eye of each rabbit. For each eye (right and left), two disks were put in an Eppendorf tube (1.5 mL) which contains 500 μL Sabouraud dextrose broth (SDB) inoculated with 10% v/v *Candida* suspension (10^7^ CFU/mL). The other two disks were put in another Eppendorf tube containing 500 μL uninoculated SDB; this was used as a blank during measuring the optical densities. All the Eppendorf tubes were then incubated at 25 ± 2 °C for 24 hours. After incubation, 200 mL from each tube was transferred to sterile 96-well plates and the optical densities (OD_600nm_) were measured using an automated spectrophotometric plate reader (Biotek, Synergy 2, USA) at a single wavelength of 600 nm. The results were presented as average percentage growth inhibition (mean ± standard deviation). The growth inhibition % was calculated using the following equation (Abdelbary et al., 2016; Fahmy et al., 2021):

(6) $$\text{Growth inhibition \%}=\frac{\text{Control Left Eye }({\mathrm{OD}}_{600nm})\;-\text{ Test Right Eye }({\mathrm{OD}}_{600nm})\;}{\text{Control Left Eye }({\mathrm{OD}}_{600nm})\;}.100$$

The area under the curve from 1 to 10 h (AUC_(1–10h)_) was calculated from the curve of each individual animal using GraphPad Prism 7 software. Student’s *t*-test was utilized to compare between the FTN-loaded optimum formula and FTN suspension.

#### 2.3.8. Statistical analysis

All factors were analyzed using Design-Expert software version 7 (Stat-Ease Inc., Minneapolis, USA) (Ahmed et al., 2021). All investigations were measured three times and outcomes were shown as mean ± standard deviation (SD). Means were compared by ANOVA analysis where significance of differences was estimated at (*p <* 0.05). One-way ANOVA was utilized for two independent groups comparisons were proper.

## 3. Results and discussions

### 3.1. Analysis of Central composite design

Central composite designs could be used to detect the influence of the independent variables: span 80 amount (mg) (A), oleylamine concentration (mg%) (B) and oleic acid: drug ratio (C) on the properties of the studied olaminosomal system (Sayed et al., 2018). The resulted responses were fitted to several order models and the predicted R^2^ values and adjusted R^2^ were in sensible harmonization for all the considered responses as presented in Table 3.

**Table 3.** Model analysis for studied responses.

Response	R^2^	Adjusted R^2^	Predicated R^2^	Adequate precision	Significant factors
EE %	0.9248	0.9107	0.8725	27.087	A,B,C^2^
PS (nm)	0.7448	0.6970	0.5205	13.810	A,C,C^2^
ZP (mV)	0.9298	0.9166	0.8557	28.035	B,C,C^2^
Q10h (%)	0.8939	0.8814	0.8642	27.280	A,B

Abbreviations: EE %, percent entrapment efficiency; PS, particle size; Q10h, percent drug released after 10 hours; ZP, zeta potential. Abbreviations: EE %, percent entrapment efficiency; PS, particle size; Q10h, percent drug released after 10 hours; ZP, zeta potential.

#### 3.1.1. Effect of studied variables on EE %

**Figure 1.**
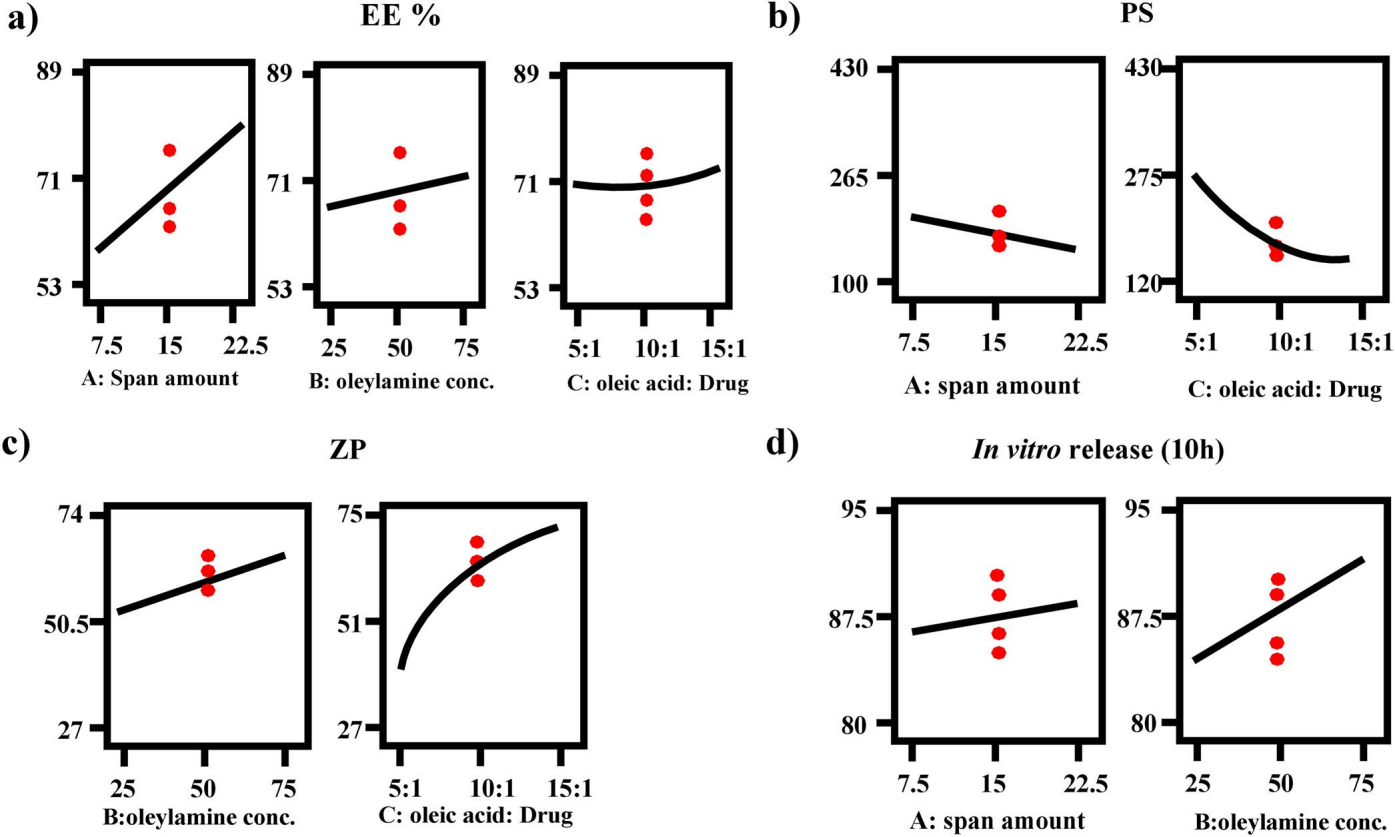
Response-plots for the effect of factor A: span amount (mg), factor B: oleylamine concentration (mg%) and factor C: oleic acid: drug ratio on (a) % EE, (b) PS, (c) ZP and (d) Q10h (%).

High EE % is a critical parameter to ensure the delivery of accepted amount of FTN for the management of ocular fungal infection (Albash et al., 2021). Results of EE % fluctuated between (53.33 ± 1.96 and 88.26 ± 0.29%), as shown in Table 2. ANOVA analysis revealed that factor A (span 80 amount (mg)), factor B (oleylamine concentration (mg%)) and factor C (oleic acid: drug ratio) had a positive significant effect (*p* < 0.05). The effects of all factors are graphically illustrated in Figure 1(a). The resulting equation in terms of coded factors was as follows:

$$\text{EE}\;\%\;=\;69.99\;+\;11.19A\;+\;2.77B\;+\;2.{39\;C}^{2}$$

Regarding factor A (*p* < 0.0001), the longer the fatty acid chains in the surfactant, the lower the HLB value. Span 80 is a surface-active agent with low HLB (4.3) as a result of its long alkyl group (oleate moiety) (Abdelbary et al., 2017). As a general rule, the lower the HLB value the higher the apparent solubility of the lipophilic drug since it would reduce the formation of hydrophilic holes and minimize the bilayers amphiphilic property (Abdelbari et al., 2021; Albash et al., 2021). It is clear that the increase of low HLB SAA (span 80) from 7.5 to 22.5 mg resulted in increasing the solubility of FTN in oleic acid and the volume of lipophilic bilayers that serves as shelter for the lipophilic FTN leading to high EE % (Hao et al., 2002; Abd-Elsalam & ElKasabgy, 2019).

Considering factor B (*p* = 0.0046), oleylamine (C_18_H_37_N) is a long fatty amine related to oleic acid with strong hydrophobic property due to its long alkyl chain which is favorable to the lipophilic FTN. Moreover, oleylamine is an amino-based surfactant that is classified as a low molecular weight gelling agent (Vizcarra-Pacheco et al., 2021). Its gelling properties arise from its ability to form a 3 D fibrillar structures that exists in the chiral self-assembly of amphiphilic molecules (Bajani et al., 2018). 3 D fibrillar structures results from non-covalent interaction such as electrostatic interactions, hydrogen bonding, hydrophobic interactions and vander Waals interactions. The formed fibrillary structures have the ability to entrap significant quantities of the surrounding medium. Thus, Increasing the concentration of oleylamine will lead to formation of more 3 D fibrillar structures, therapy EE % increases (Fan et al., 2021).

Regarding factor C (*p* = 0.0094), oleic acid (C_18_H_34_O_2_) is a long chain fatty acid with high hydrophobic characters due to its long alkyl chain. Generally, increasing the hydrophobicity of the medium would results in significant entrapment of the lipophilic FTN (Ahmad et al., 2019). Increasing the ratio of oleic acid would increase its concentration which would increase the solubility of lipophilic FTN in the lipid bilayers (Mishra et al., 2022). Moreover, increasing the amount of oleic acid would enhance the capping effect of oleylamine as a result of formation of more carboxylate derivatives that yields from the interaction between COOH of oleic acid and NH group of oleylamine (Safo et al., 2019).

#### 3.1.2. Effect of studied variables on PS

The particle size (PS) of the formed olaminosomes is a critical property to detect their suitability for ocular delivery, as it effects their tolerance and disposition in the eye (Younes et al., 2018b). PS ranged from (143.00 ± 2.26 to 427.35 ± 78.14 nm), as shown in Table 2. ANOVA analysis revealed that factor A (span 80 amount (mg)), and factor C (oleic acid: drug ratio) had a negative significant effect (*p* < 0.05). However, factor B (oleylamine concentration (mg%)) had non-significant effect (*p* > 0.05). The outcomes of these factors are shown in Figure 1(b). The resulting equation in terms of coded factors was as follows:

$$\text{PS}\;=\;170.22\;–\;25.62A\;–\;49.54C\;+\;29.{56C}^{2}$$

Oleylamine is a strong capping agent that could attach tightly to the surface of the formed olaminosomes. It has a considerable steric hindrance effect resulted from its long chain alkyl group that could control the size of the formed nanoparticles. Surface adsorption of oleylamine also helps to accomplish size uniformity, avoid large agglomeration and fuzing of particles (Kumar et al., 2014).

Considering factor A (*p* = 0.0093), increasing the amount of surfactant (span 80) permits additional coverage for the surface of olaminosomes resulting in reducing the interfacial tension between the formed olaminosomes and the aqueous surrounding, thus preventing the aggregation of olaminosomes and reducing the PS (ElKasabgy et al., 2014). Similar results were previously mentioned by Eldeep et al. working on formulation and evaluation of cubosomes of Brimonidine tartrate for treatment of glaucoma (Emad Eldeeb et al., 2019). Also, increasing the amount of surfactant will lead to the formation of more olaminosomes that would be able to entrap FTN inside the core without excessive core swelling, thus PS decreases. Similar results were formerly discussed by Younes et al. working on ocular delivery of solutol HS15 based binary mixed micelles of sertaconazole nitrate (Younes et al., 2018b).

Regarding factor C (*p* = 0.0007), oleic acid is a penetration enhancer that has a certain effect on size, elasticity and permeability (Abdulbaqi et al., 2016). The existence of permeation enhancer would destabilize the lipid bilayer of the formed olaminosomes by reducing its surface tension and rigidity, thus reducing PS (Manconi et al., 2009). Similar results were previously mentioned by Song et al. working on transethosome for enhanced skin delivery of voriconazole (Song et al., 2012).

#### 3.1.3. Effect of studied variables on PDI

Considering PDI, a value of 0 indicates homo-dispersed system, while a value of 1 indicates highly poly-dispersed system (Mosallam et al., 2021). PDI of the prepared FTN-loaded formulae varied from (0.08 ± 0.10 to 0.41 ± 0.03) (Table 2). These outcomes ensured the accepted homogeneity of the formulae. ANOVA analysis indicated that all the studied variables had a non-significant effect on PDI (*p* > 0.05). So it was excluded from optimization criteria.

#### 3.1.4. Effect of studied variables on ZP

ZP is used to detect the total surface charge of the constructed formulae to detect its physical stability and predict any potential interaction inside the body. High absolute values of ZP signify better surface charge, minor particle-particle interaction and superior physical stability (Albash et al., 2021). ZP varies from (−27.10 ± 0.85 to −73.80 ± 4.38) ensured that all formulae had enough charge to avoid aggregation of particles. ANOVA analysis showed that factor B (oleylamine concentration (mg%)) and factor C (oleic acid: drug ratio) had a positive significant effect (*p* < 0.05). However, factor A (span 80 amount (mg)) had non-significant effect (*p* > 0.05) since span 80 is a non-ionic surfactant that does not impart any charge on nanoparticles. The effects of all factors are graphically explained in Figure 1(c). The resulting equation in terms of coded factors was as follows:

$$\text{ZP}\;=\;59.31\;+\;4.35B\;+\;16.42C\;–\;3.{96C}^{2}$$

Oleic acid (C_17_H_33_COOH) has a free carboxylic group that would significantly increase (*p* < 0.0001) the negative charge of the formed olaminosomes as a result of ionization (Abd-Elsalam & ElKasabgy, 2019). There is an inverse relationship between ZP and aggregation of particles, since aggregation of particles would reduce the stability and ZP of the formed system. Generally, aggregates are formed when ZP is low since the attraction forces will overcomes repulsion forces. So, ZP is used to predict the possibility of aggregate formation (Samimi et al., 2019). Oleylamine is a well-known capping agents which is adsorbed on the surface of nanoparticles preventing over-growth and aggregation of the resulted nanoparticles therefore affecting the stability of the formed system (Javed et al., 2020). Presence of Oleylamine would decreases further interaction of carboxylic groups of oleic acid with amino group of oleylamine in neighbored particles. Oleylamine has a high proton affinity that significantly (*p* = 0.0023) stabilizes the interface between the formed olaminosomes and the surrounding medium (Mbewana-Ntshanka et al., 2020). We can conclude that the significant capping effect of oleylamine will increase ZP as a result of increased stability and aggregation prevention.

#### 3.1.5. Effect of studied variables on *in vitro* release (Q10h)

**Figure 2.**
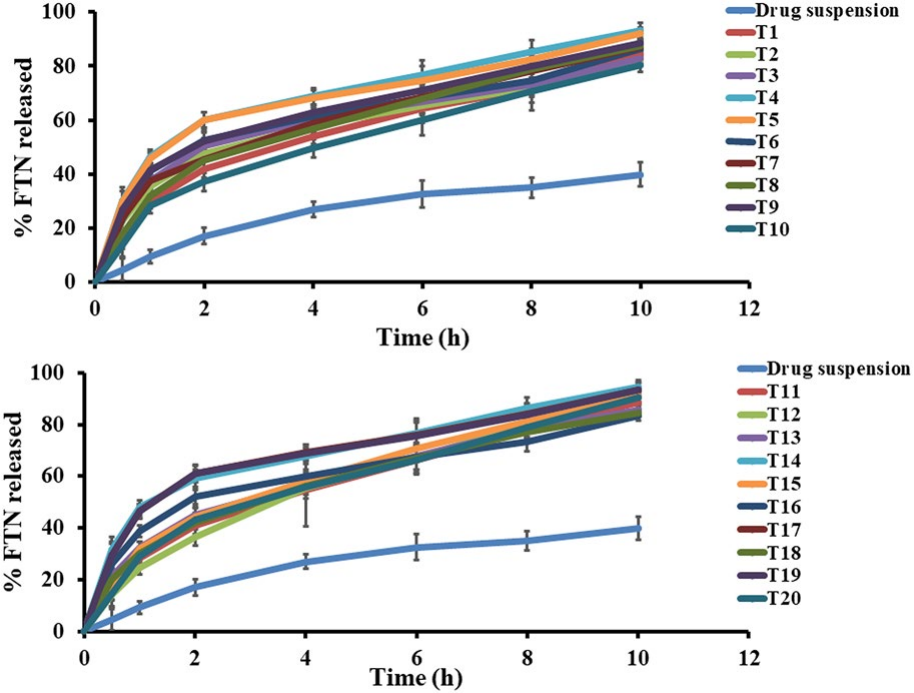
*In vitro* Fenticonazole (FTN) release profiles from investigated formulae and drug suspension at 37 ± 0.5 °C, mean ± SD, *n* = 3.

In order to differentiate between the different prepared FTN-loaded formulae, *in vitro* release study was done compared to the release of FTN from its suspension. Q10h of all the constructed formulae varied from (80.37 ± 2.49 to 94.44 ± 2.61) as shown in Table 2 and illustrated in Figure 2. Constructed formulae have significantly quicker (*p <* 0.05) release profiles than the corresponding FTN suspension. This performance was principally attributed to the smaller particle size of the formed olaminosomes when matched to the size of the pure drug suspension (coarse dispersion). Concerning ANOVA analysis, factor A (span 80 amount (mg)) and factor B (oleylamine concentration (mg%)) had a positive significant effect (*p <* 0.05). While factor C (oleic acid: drug ratio) had non-significant effect (*p >* 0.05) on Q10h. The results of these factors are revealed in Figure 1(d). The resulting equation in terms of coded factors was as follows:

Q10h = 87.98 +1.15A + 4.58B

Concerning the significant effect of span 80 concentration (factor A) with *p value* (0.0097), this might be linked to the enhanced solubility of FTN and the reduced PS resulted from incorporation of span 80. Incorporation of span 80 results in increasing the solubilization of FTN in oleic acid between the hydrophobic moieties of lipid bilayers resulting in smaller PS, thereby faster release (Abd-Elsalam & ElKasabgy, 2019; Abd-Elsalam & Ibrahim, 2021). Factor B significantly (*p* < 0.0001) decrease the interfacial tension as a result of the capping effect of oleylamine that would increase in affinity of FTN to release medium, thereby high release. In addition, the effect of oleylamine prevents particles aggregation resulting in smaller PS. thereby rapid release.

The release profiles of the prepared FTN-loaded olaminosomes could be divided into two steps. The first rapid step due to the existence of some unentrapped FTN close to the surface attached to the long hydrocarbon chain in the lipid bilayer followed by a retarded step due to the high association of the lipophilic FTN within the core of the prepared olaminosomes (Abd-Elsalam & Ibrahim, 2021). Higuchi-diffusion model is the most appropriate model to rationalize the release profiles (highest r-square) (Ahmed et al., 2021).

### 3.2. Selection of the FTN-loaded optimum formula

The optimum levels of the studied variables were selected by analysis of the results of dependent variables using Design-Expert^®^ software to choose the FTN-loaded optimum formula with the greatest EE %, ZP and Q10h, in addition to the smallest probable PS and PDI. The software chose the formula with the largest desirability factor (0.972) which was constructed using (span 80 amount = 22.50 mg, oleylamine concentration= 74.99 mg and oleic acid: drug ratio = 14.90). The FTN-loaded optimum formula possessed EE % = 84.24 ± 1.28%, PS = 117.55 ± 5.44 nm, ZP = −74.85 ± 1.91 mV and Q10h = 91.26 ± 0.96%. The predicted results of the dependent variables were in good agreement with the actual values, as presented in Table 4 ensuring the fitness of the statistical design (Yousry et al., 2020).

**Table 4.** Characterization of the optimum formula.

Response	Y1	Y2	Y4	Y5
	EE %	PS (nm)	ZP (mV)	Q10h (%)
Observed value	84.24	117.55	−74.85	91.26
Predicated value	86.24	115.12	−75.94	93.72
% Deviation (absolute)	2.32	2.11	1.44	2.63

Abbreviations: EE %, percent entrapment efficiency; PS, particle size; Q10h, percent drug released after 10 hours; ZP, zeta potential.

### 3.3. In vitro characterization of the FTN-loaded optimum formula

#### 3.3.1. Differential scanning calorimetry (DSC)

**Figure 3.**
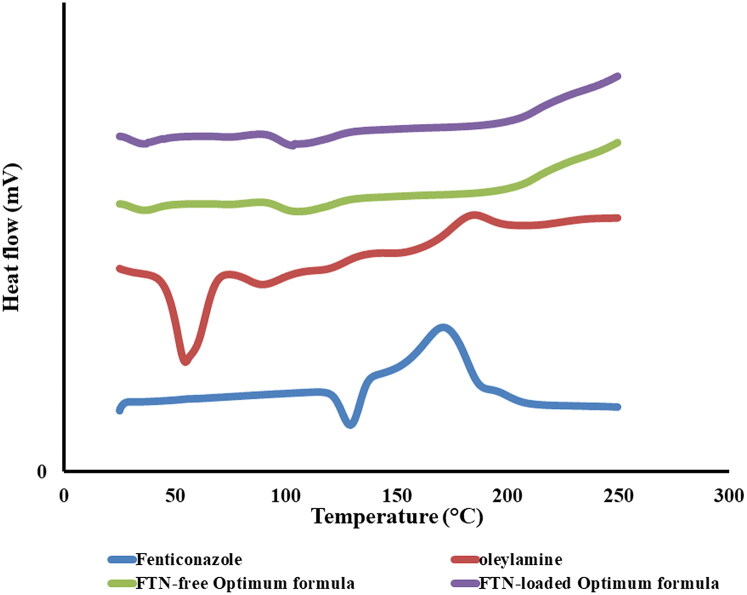
DSC thermogram of pure Fenticonazole (FTN), oleylamine, FTN-loaded optimum formula and FTN-free optimum formula.

DSC thermograms of FTN, oleylamine, lyophilized FTN-loaded and FTN-free optimum formulae are shown in Figure 3. The characteristic endothermic peak of FTN that is related to its melting point was shown at 135 °C (Albash et al., 2020). Oleylamine thermogram showed an endothermic peak at 54.59 °C. The optimum formulae did not reveal the characteristic peak of FTN as a result of the encapsulation of FTN in the vesicles (Salih et al., 2020).

#### 3.3.2. Fourier transforms infrared spectroscopy (FTIR)

**Figure 4.**
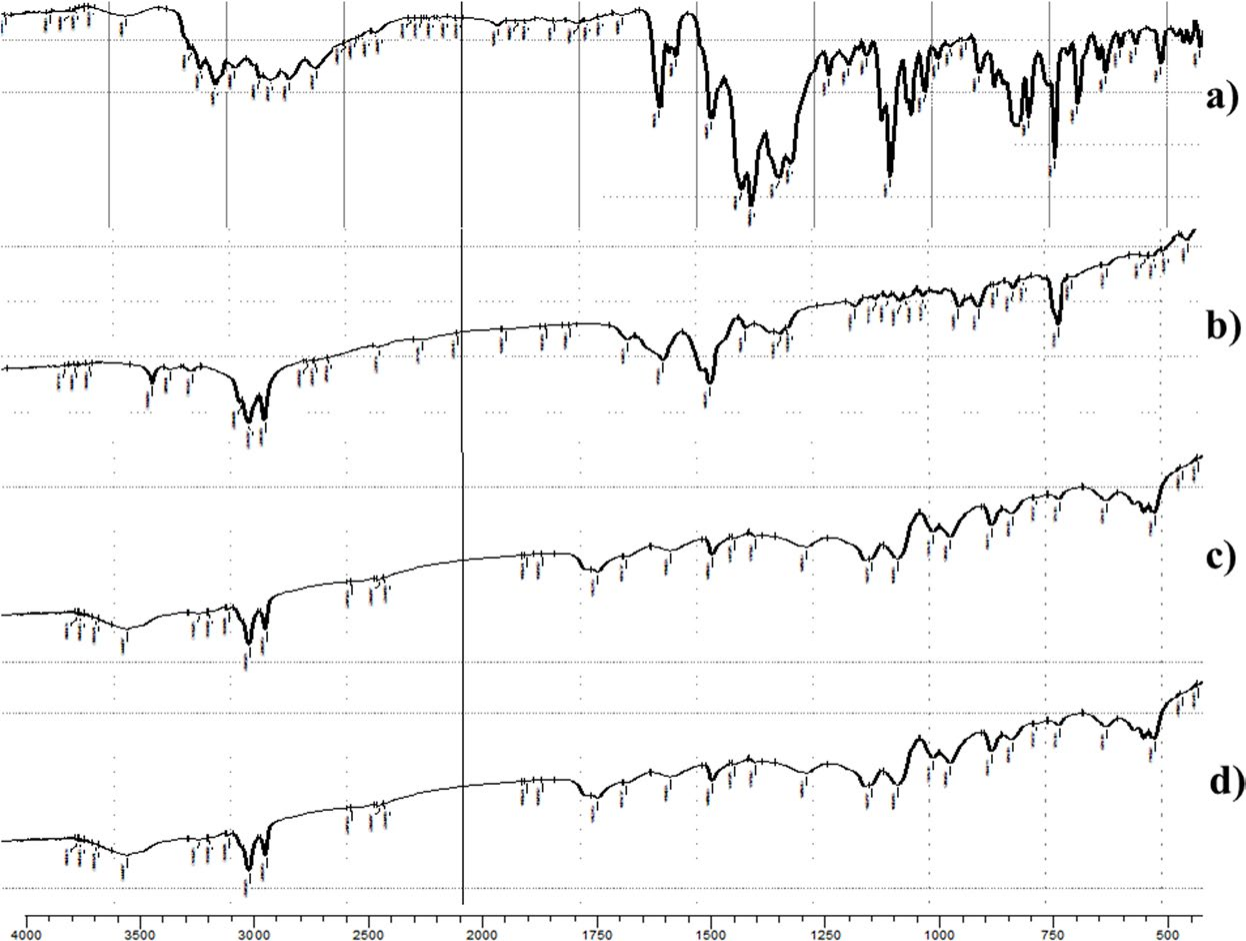
The FTIR spectra of (a) pure Fenticonazole (FTN), (b) oleylamine, (c) FTN-loaded optimum formula and (d) FTN-free optimum formula.

FTIR spectra of FTN, oleylamine, lyophilized FTN-loaded and FTN-free optimum formulae are shown in Figure 4. Fenticonazole nitrate has a clear structure resemblance with chlorinated imidazoles (Castro et al., 2016). The FTIR spectra of FTN are shown at 1581.63, 1469.76, 1091.71 and 794.67 cm^−1^ related to C = N stretching, C = C aromatic stretch, C-O-C ether stretch and C-Cl stretch respectively (Castro et al., 2016). The characteristic peaks of oleylamine are revealed at 3332.99, 2860.79 and 2916.37 cm^−1^. The peak at 3332.99 cm^−1^ is correlated to N-H bond stretching. However, peaks at 2860.79 and 2916.37 cm^−1^ are linked to C-H stretching (Ranjith Kumar et al., 2013). The optimum formulae revealed the characteristic peaks of amide group that results from the interaction between the amino group of oleylamine and the carboxylic group of oleic acid. Generally, amide group reveals the characteristic peaks of both N-H bond and C = O bond. The optimum formulae revealed a strong band at 3332.99 cm^−1^ that is related to N-H bond stretching (Amide A peak). Also, it revealed stake-shaped band around 1710 cm^−1^ for the C = O stretch (Amide I peak) (Durukan et al., 2019; Ji et al., 2020). These results demonstrated the significant capping effect of oleylamine. Complete Entrapment of FTN was confirmed by the absence of its characteristic peaks from the spectra of FTN-loaded optimum formula (Ahmed et al., 2020). The outcomes of FTIR came in harmony with that obtained from DSC.

#### 3.3.3. TEM microscopy

**Figure 5.**
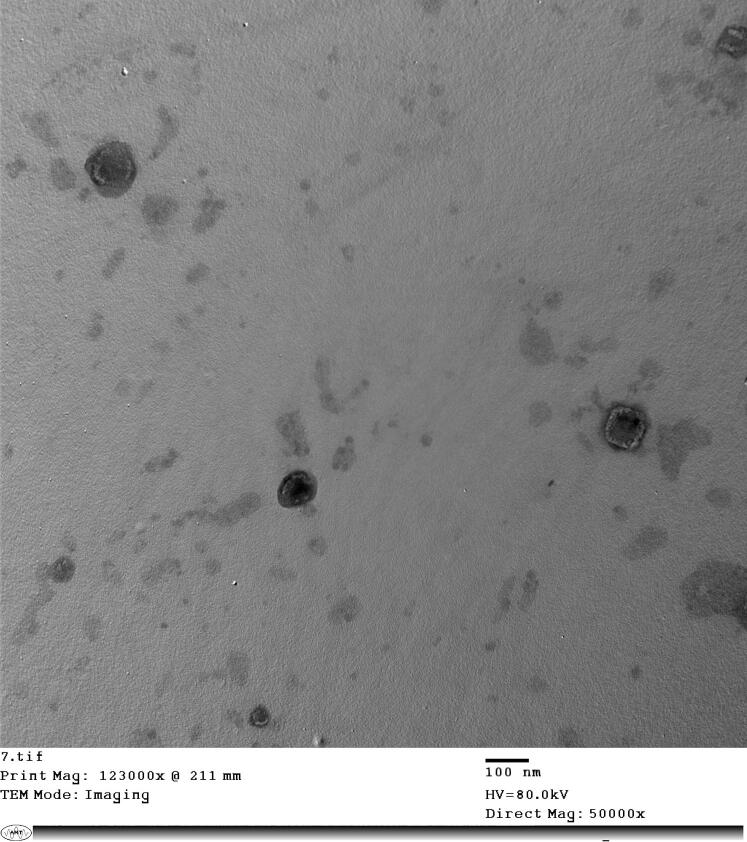
TEM of the optimum formula.

TEM of the FTN-loaded optimum formula was shown in Figure 5. TEM proved the characteristic spherical, non-accumulated with a smooth surface and minute size olaminosomes. TEM demonstrated the significant capping effect of oleylamine since it was adsorbed on the surface of nanoparticles maintaining mono-dispersity and preventing over-growth and aggregation of the resulted nanoparticles. These images were in harmony with the previously mentioned results by Abd-Elsalam and ElKasabgy (Abd-Elsalam & ElKasabgy, 2019).

#### 3.3.4. pH measurement

Since eye is a very sensitive organ, thereby pH measurement is particularly significant to avoid any chemical eye damage after instillation and to ensure effective permeation. pH of the FTN-loaded optimum formula was 7.34 ± 0.04, which is regarded to be safe (Said et al., 2021). Similar results were previously mentioned by Sayed et al. (2021)

#### 3.3.5. Effect of short-term storage

The FTN-loaded optimum formula was preserved for 3 months at (4–8 °C) with regular visual inspection. The preserved formula did not reveal any aggregation or alteration in its physical appearance. Furthermore, EE %, PS and PDI measurements showed insignificant difference (*p* > 0.05), as shown in Table 5 (Albash et al., 2021). The similarity factor (ƒ_2_) was equal to 77.52, ensuring similar *in vitro* release profiles (Sayed et al., 2020; 2021). These results ensures the high stability of the FTN-loaded optimum formula that might be resulted from its unique structure. Presence of oleic acid provides high negative charge (ZP = −74.85 ± 1.91 mV). Oleylamine has a pronounce capping effect that stabilizes the surface of the formed vesicles preventing its aggregation and thereby increase its stability after storage. Also, the minute PS would allow further exposure of the ionizable groups due to the increased surface area. Finally stearic stabilization caused by the large structure of oleylamine and oleic acid (Nguyen et al., 2009; Abd-Elsalam & ElKasabgy, 2019).

**Table 5.** Effect of short-term stability and gamma sterilization on the optimum formula.

		Storage for 3 months at 4–8 °C		After gamma sterilization	
Parameter	Fresh	Value	Probability (*p*)*	Value	Probability (*p*)**
EE %	84.24 ± 1.28	80.35 ± 4.62	0.370	83.26 ± 1.47	0.555
PS	117.55 ± 5.44	124.15 ± 2.90	0.269	117.85 ± 6.29	0.964
ZP	−74.85 ± 1.91	−74.15 ± 0.21	0.658	−69.45 ± 3.89	0.220

Abbreviations: EE %, percent entrapment efficiency; PS, particle size; ZP, zeta potential.

* One-way ANOVA analysis to compare between the freshly prepared and the stored optimum formula.

** One-way ANOVA analysis to compare between the freshly prepared and gamma sterilized optimum formula.

#### 3.3.6. Effect of terminal sterilization

Sterilization of the final ocular dosage form is a must to avoid co-infecting with hazardous microbes (Emad Eldeeb et al., 2019). The visual inspection of the sterilized formula revealed no alteration in the physical appearance. The percent EE, PS and ZP values revealed non-significant change (*p* > 0.05), as shown in Table 5. The similarity factor (ƒ_2_) was equal to 83.19, ensuring similar *in vitro* release profiles (Sayed et al., 2020; 2021). Therefore, gamma sterilization could be used safely to disinfect the FTN-loaded optimum formula.

#### 3.3.7. Minimum inhibitory concentration (MIC) determination

The antifungal activity of the optimized FTN-loaded olaminosomes and FTN suspension were evaluated *in vitro* using *Candida albicans* ATCC 60193 as the test organism. MIC for the optimized FTN-loaded olaminosomes was found to be equal to 62.5 ug/mL compared to FTN suspension which was higher than 500 ug/mL. The optimized FTN-loaded olaminosomes showed more than 3 times higher the antifungal activity than that of the FTN suspension, indicating that the incorporation of FTN within the olaminosomes enhanced its *in vitro* antifungal activity.

The superiority of the antifungal activity of the FTN-loaded optimum formula over FTN suspension might be due to the capping effect of oleylamine that results in small particle size and high zeta potential value in addition to the presence of oleic acid as a penetration enhancer. The capping effect of oleylamine prevents the aggregation of the formed olaminosomes leading to smaller PS that is useful in membrane penetration of antifungal drugs. ZP plays an important role in the interaction between the formulated vesicles and the cationic sites present on microbial cell surface that allow nonspecific adsorption of the negatively charged particles in the form of clusters resulted from the repulsive interactions between olaminosomes and the large negatively charged domains of the cell surface. Additionally, the adsorbed particles would develop a reduced charge density that would encourage further adsorption of other free particles (Patil et al., 2007). Oleic acid has fixed bend C = C bonds that inhabit a wider cross section upon entering the fungal membrane, thus improving the oxidative stress and fungicidal activity of the formed olaminosomes (Mosallam et al., 2021). In conclusion, the FTN-loaded optimum formula had a higher antifungal activity and lower MIC compared to FTN suspension.

### 3.4. Ex vivo characterization of the FTN-loaded optimum formula

#### 3.4.1. *Ex vivo* corneal penetration

**Figure 6.**
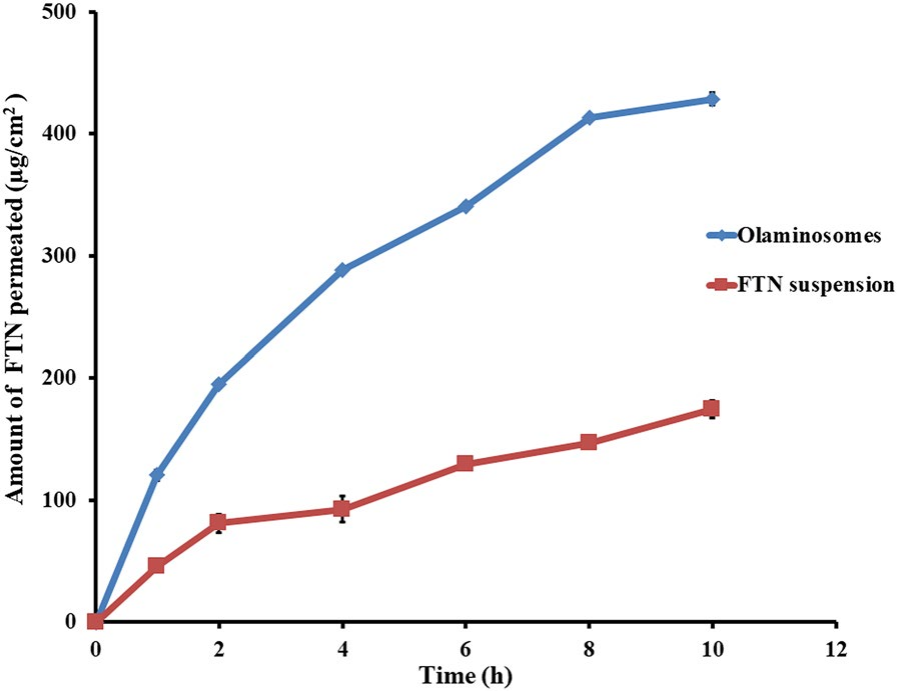
The *ex-vivo* permeation profiles of the optimum formula compared to that from FTN suspension at 37 ± 0.5 °C, mean ± SD, *n* = 3.

The penetration profiles of the FTN-loaded optimum formula and FTN suspension are shown in Figure 6. The FTN-loaded optimum formula showed augmented penetration after 10 h (Q_10h-permeation_ of the FTN-loaded optimum formula = 428.66 ± 4.86 µg/cm^2^) compared to (Q_10h-permeation_ of FTN suspension = 174.66 ± 6.94 µg/cm^2^). The FTN-loaded optimum formula also showed greater flux (J_max_ of the FTN-loaded optimum formula = 42.87 ± 0.49 µg/cm^2^/h) compared to (J_max_ of FTN suspension = 17.47 ± 0.69 µg/cm^2^/h). One-way ANOVA verifies that the FTN-loaded optimum formula had significantly higher penetration after 10 h and flux (*p* < 0.05) compared to FTN suspension with enhancement ratio of 2.45. The higher penetration results of olaminosomes might be due to the capping effect of oleylamine that results in small particle size and high zeta potential, which assist drug permeation (Younes et al., 2018b; Mosallam et al., 2021). Also, span 80 might enhance the penetration by softening the rigid connections of the corneal epithelium (Li et al., 2014). Finally, the presence of oleic acid as penetration enhancer would increase the elasticity and permeability of the formed vesicles by reducing its surface tension and rigidity and finally destabilizing the lipid bilayer (Manconi et al., 2009; Abdulbaqi et al., 2016).

#### 3.4.2. *Ex vivo* corneal hydration level

The corneal hydration level (%HL) was determined to detect any damage to the corneal tissues after the *ex vivo* study. The normal healthy cornea has %HL ranged from (76–80%) (Dai et al., 2013). HL % of FTN-loaded optimum formula and FTN suspension were (77.58 ± 1.27 and 79.25 ± 0.48 correspondingly). One-way ANOVA test demonstrated the absence of significant difference (*p* > 0.05) in % HL obtained from the FTN-loaded optimum formula and FTN suspension. Accordingly, olaminosomes might be regarded harmless to the eye (Younes et al., 2018b).

### 3.5. In vivo characterization of the FTN-loaded optimum formula

#### 3.5.1. *Ocular* irritancy test

No signs of inflammation or boosted tear formation were noticed for 24 h. Therefore, olaminosomes could be regarded safe and harmless to the eye (Abdelbary et al., 2017).

#### 3.5.2. *In vivo* corneal tolerance

**Figure 7.**
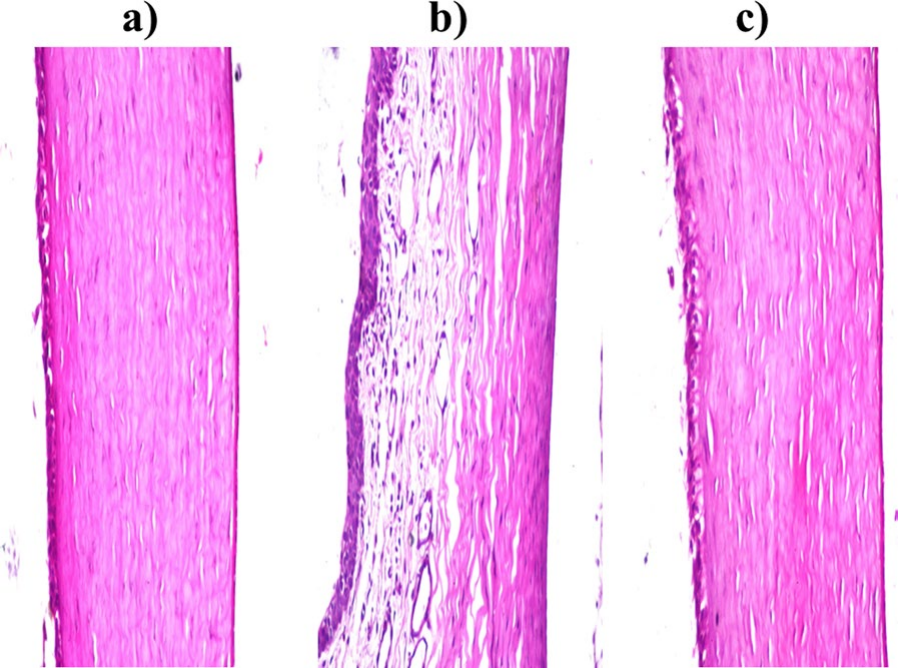
Photomicrographs of the rabbits’ corneas after instillation of; a) Normal saline solution (negative control), b) Isopropyl alcohol (positive control) and c) Optimum formula.

Regarding corneal tissues treated with normal saline as negative control; no histopathological alteration in the covering epithelium as well as the underlying stroma and endothelium, as shown in Figure 7(a). Concerning corneal tissues treated with isopropyl alcohol as positive control; The covering epithelial layer showed focal stratification associated with edema and inflammatory cells infiltration in the underlying stroma, as shown in Figure 7(b). Finally, corneal tissues treated with the FTN-loaded optimum formula; no histopathological alteration as recorded in Figure 7(c). Thus, olaminosomes could be administrated to the eye without harmful consequences (Albash et al., 2021).

#### 3.5.3. *In vivo* corneal uptake

**Figure 8.**
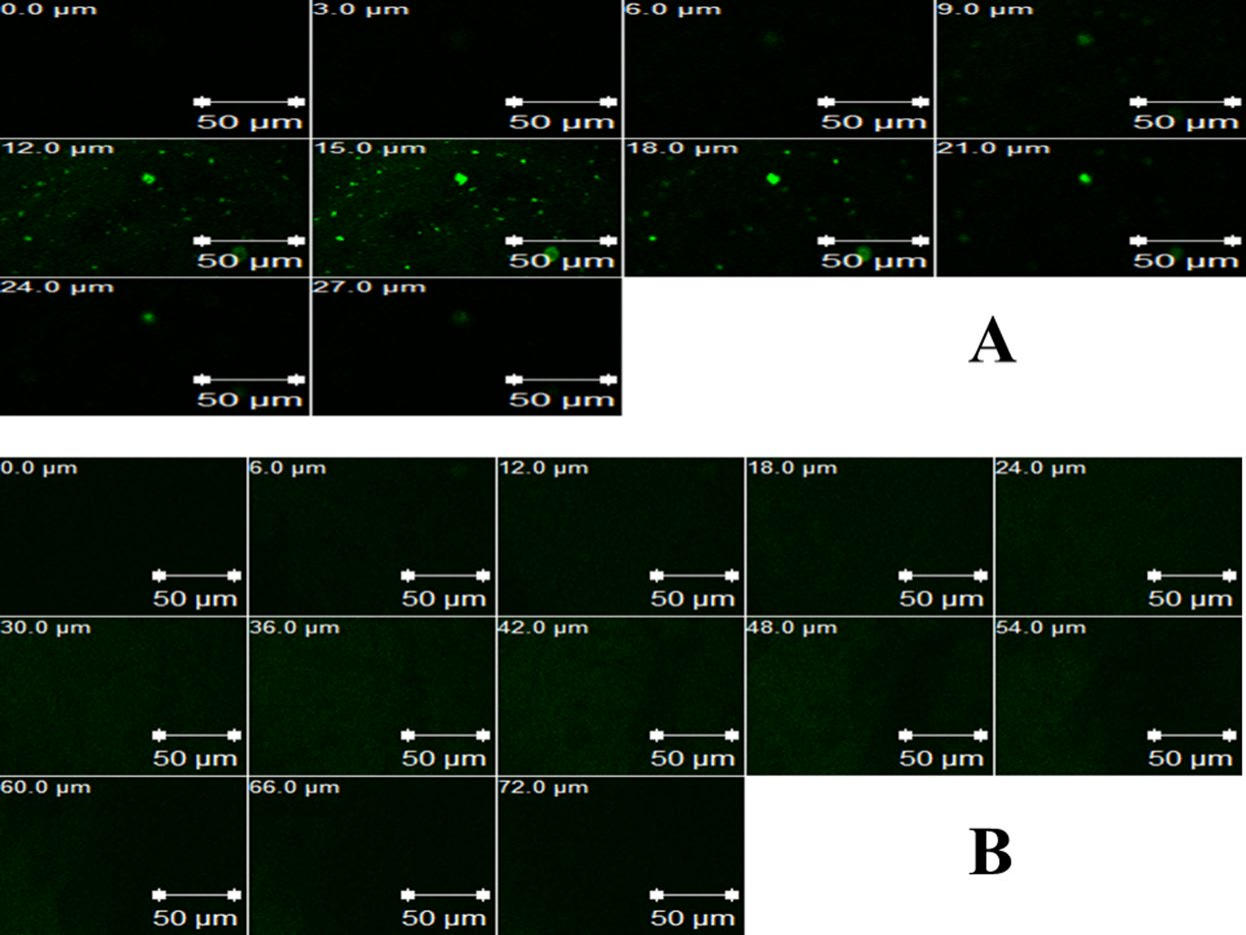
Confocal laser scanning micrographs of rabbit corneas after instillation of: a) RhB-loaded aqueous solution and b) RhB-loaded optimum formula.

In order to confirm the capability of the FTN-loaded optimum formula to improve the corneal penetration of FTN, CLSM was used to distinguish the transcorneal performance of RhB-loaded formulae after instillation, by tracking the fluorescence signals inside the corneal tissues. CLSM micrographs show greater penetration from RhB-loaded olaminosomes (72 μm) than RhB-loaded aqueous solution (27 μm), as presented in Figure 8. Similar results were previously obtained from *ex vivo* penetration study. It is noted that higher delivery of the antifungal drug (FTN) is required, as it represents a suitable therapy for deep fungal ocular diseases (Sayed et al., 2018; Younes et al., 2018b).

#### 3.5.4. Susceptibility test

**Figure 9.**
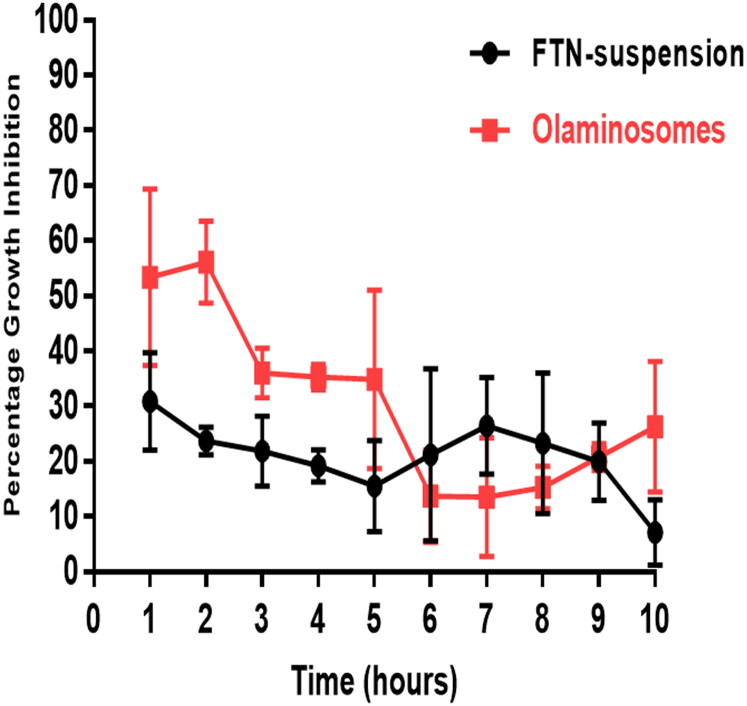
Percentage inhibition of *Candida albicans* growth produced by FTN-loaded optimum formula and the FTN suspension in rabbit external ocular tissue.

The antifungal activity of the FTN-loaded optimum formula was compared to that of FTN suspension following *in vivo* animal model using *Candida albicans* ATCC 60193 as the test organism. The percentage growth inhibition of *Candida albicans* produced by the different formulae was plotted against time. Figure 9 shows that the percentage growth inhibition from the FTN-loaded optimum formula reached the maximum (56.13 ± 7.4%) two hours post-administration and then decreased gradually. On the other hand, FTN suspension reached a maximum of 30.83 ± 8.8% one-hour post-administration and then showed almost constant level from the second hour until the ninth hour of the study period (27 ± 2.5% − 20.16 ± 7.02% from 2 h − 9 h). The growth inhibition percentage of the FTN-loaded optimum formula was significantly higher than that of the FTN suspension until the fifth hour post-application (Student’s *t*-test, *p* < 0.05, *p-*value =0.0048).

The FTN-loaded optimum formula significantly sustained the antifungal activity of FTN on the ocular surface for a relatively longer time when compared to the FTN suspension with an area under the curve 1.3 folds higher than that of FTN suspension (AUC1h-10h = 265.5 ± 19.36% and 190.2 ± 18.9%, respectively). This probably was due to the high solubilization of FTN inside the core and between the amphiphilic lipid bilayers leading to high amount of incorporated FTN either in the core or in between the oleic acid bilayers giving rise to more sustainment of FTN effect. However, the enhanced permeation properties were due to the presence of span 80 that would soften the rigid connections of the corneal epithelium (Li et al., 2014). In addition to, the presence of oleic acid as a penetration enhancer results in higher corneal permeation and provides a high negative charge. Furthermore, the capping effect of oleylamine would prevent the aggregation of nanoparticles that resulted in efficient stabilization and enhanced antimicrobial activity (Javed et al., 2020).

## 4. Conclusions

In this study, olaminosomes were successfully formulated by ethanol injection method. The FTN-loaded optimum formula evidenced small particle size (117.55 ± 5.44 nm), high percent entrapment efficiency (84.24 ± 1.28%), acceptable zeta potential (−74.85 ± 1.91 mV), great % *in vitro* release (Q10h) (91.26 ± 0.96%), spherical morphology and excellent physicochemical stability. The fulfilled entrapment of FTN within the FTN-loaded optimum formula was proved through DSC and FTIR studies. FTIR studies also confirmed the formation of amide group that demonstrates the capping effect of oleylamine. It has been clarified that the FTN-loaded optimum formula had achieved improved *ex vivo* corneal permeation (428.66 ± 4.86 µg/cm^2^) compared to FTN suspension (174.66 ± 6.94 µg/cm^2^) and high stability following gamma irradiation sterilization. Safety of the FTN-loaded optimum formula was evidenced via pH measurement, *ex vivo* corneal hydration level, *in vivo* ocular irritancy test and *in vivo* corneal tolerance test. *In vitro* MIC determination, *in vivo* corneal uptake and susceptibility test were used to ensure both *in vitro* and *in vivo* fungal activity of the FTN-loaded optimum formula. Finally, olaminosomes could be considered as promising nano-carriers for enhancing the ocular delivery of the antifungal Fenticonazole nitrate.
